# Strength of the Ancients

**Published:** 1884-05

**Authors:** 


### GREAT STRENGTH OF THE ANCIENTS.

PHYSICAL superiority of the ante-Alexandrian Greeks to the hardiest and most robust nations of modern times, is perhaps best illustrated by the military statistics of Xenophon. According to the author of the "Anabasis," the complete accoutrements of the Spartan soldier, in what we would call heavy marching order, weighed seventy-five pounds, exclusive of the camp, mining, and bridge-building tools, and the rations of bread and dried fruit which were issued in weekly instalments and increased the burden of the infantry soldier to ninety, ninety-five, or even to a full hundred pounds. This load was often carried at the rate of four miles an hour for twelve hours per diem, day after day; and only in the burning deserts of southern Syria the commander of the Grecian auxiliaries thought it prudent to shorten the usual length of a day's march.

